# Genetic variability in fruit-to-bean conversion efficiency among *Coffea canephora* genotypes

**DOI:** 10.3389/fpls.2026.1878674

**Published:** 2026-07-13

**Authors:** Alex Campanharo, Deurimar Herênio Gonçalves Júnior, Marcela Campanharo, Maskio Darós, Adésio Ferreira, Weverton Pereira Rodrigues, Henrique Duarte Vieira, Isabel Marques, Fábio Luiz Partelli

**Affiliations:** 1 Federal University of Espírito Santo, University Center of Northern Espírito Santo, São Mateus, Espírito Santo, Brazil; 2 Technical Assistance and Rural Extension Company of the State of Minas Gerais, Pocrane, Minas Gerais, Brazil; 3 Center for Agricultural Sciences, State University of the Tocantina Region of Maranhão, Imperatriz, Maranhão, Brazil; 4 Laboratory of Crop Science, Center for Agricultural Sciences and Technologies, State University of Northern Rio de Janeiro Darcy Ribeiro, Campos dos Goytacazes, Rio de Janeiro, Brazil; 5 Forest Research Center, Associate Laboratory TERRA, School of Agriculture, University of Lisbon, Lisboa, Portugal

**Keywords:** bean-to-husk ratio, genetic variability, plant breeding, robusta coffee, yield efficiency

## Abstract

**Introduction:**

*Coffea canephora* plays a strategic role in global coffee production and is increasingly cultivated in non-traditional regions as climate change reshapes suitable growing areas. Understanding genotype performance in these new environments is essential for maintaining productivity and processing efficiency.

**Methods:**

We evaluated 44 genotypes cultivated in eastern Minas Gerais, Brazil, a recently established production frontier. Traits assessed included the ratio of ripe fruit mass to dry bean mass (RFM/DBM), the ratio of ripe fruit volume to ripe fruit mass (RFV/RFM), grain and husk mass percentages, and average yield expressed in fruit volume and mass.

**Results:**

Significant genetic variability was detected, with moderate to high heritability (48–59%). Hierarchical clustering identified four groups with contrasting efficiency profiles, while mean comparisons revealed genotypes with reduced fruit biomass requirements, higher fruit density, and greater grain proportion. Bean mass percentage ranged from 48% to 61%, and average yield varied from 325.92 to 470.33 L per 60-kg bag and from 209.22 to 282.61 kg of ripe fruits per bag. Several genotypes showed superior conversion efficiency, including LMG 11, LMG 01, LMG 07, LMG 08, and Graudão HP, which combined low fruit-to-bean ratios with high grain mass proportions.

**Discussion:**

These results demonstrate exploitable genetic variability and provide reference parameters to support breeding, regional recommendations, and strategies to enhance processing efficiency in *C. canephora* systems.

## 1 Introduction

Coffee, recognized as one of the most valuable agricultural commodities worldwide, sustains millions of producers and drives a global market estimated to exceed US$400–500 billion in the coming years, depending on market segmentation and methodology (Mordor Intelligence, 2024). Beyond beverage consumption, coffee represents a complex agro-industrial system in which production efficiency, processing yield, and raw material utilization directly affect economic performance along the value chain. Climate change, however, is reshaping traditional coffee-growing regions with increasing temperatures and altered rainfall regimes compromised productivity, particularly of the more climate-sensitive species *Coffea arabica* (Etana and Merga, 2023; Gil-Gómez et al., 2024). These challenges have accelerated the strategic importance of *Coffea canephora* (Robusta/Conilon), a species characterized by greater tolerance to heat and water deficit and higher resilience to pests and diseases, traits that reduce production risks and input dependency (Ferrão et al., 2024). Currently responsible for approximately 42.6% of global coffee production, *C. canephora* is indispensable to the soluble coffee industry and to espresso blends, reinforcing its central role in the industrial coffee sector (Gil-Gómez et al., 2024; Oliveira and Oliveira, 2023; Santiago et al., 2025).

In parallel with climate-driven shifts in suitability, *C. canephora* cultivation has expanded into new production areas previously considered marginal, including eastern Minas Gerais, Brazil (Lorençone et al., 2023). While these emerging frontiers offer opportunities to increase supply, they also introduce uncertainty regarding raw material quality and processing efficiency. Genotype performance in *C. canephora* is strongly influenced by environmental conditions, and differences in fruit physical characteristics can substantially affect downstream operations, including harvesting, transport, drying, and industrial processing (Ferrão et al., 2024; Jordaim et al., 2025). In emerging production areas, variability in fruit physical traits may introduce additional challenges for industrial integration, particularly when processing infrastructure was designed based on assumptions derived from traditional producing regions. Variations in fruit density, husk proportion, and conversion efficiency can affect transport logistics, drying schedules, and processing capacity, underscoring the importance of selecting genotypes that align with industrial requirements in addition to agronomic performance (Salvador et al., 2025). Under climate change scenarios, where heat and water stress increasingly affect fruit development, this variability in fruit biomass partitioning may be amplified, further influencing processing efficiency and industrial yield stability. Understanding how genotypes perform in such conditions is therefore critical for securing a resilient raw material supply for the coffee industry (De Aquino et al., 2022).

Among the decisive parameters for economic viability, traits related to fruit biomass partitioning directly determine the efficiency with which fresh fruits are converted into marketable dry beans. Among these, the ratio between ripe fruit mass and dry bean mass (RFM/DBM, kg kg^−1^) and the ratio between ripe fruit volume and ripe fruit mass (RFV/RFM, L kg^−1^) are particularly informative as direct indicators of yield efficiency. The RFM/DBM ratio quantifies how much fresh fruit biomass is required to produce a given mass of processed beans, directly influence processing yield, energy consumption, and waste generation. In turn, the RFV/RFM ratio reflects fruit density and husk proportion, traits that affect transport efficiency, storage requirements, and drying capacity. From an industrial standpoint, differences in fruit-to-bean conversion efficiency have cascading effects across the processing chain. Higher RFM/DBM ratios increase the amount of biomass that must be harvested, transported, and dried to produce a fixed quantity of green coffee, leading to higher energy use and operational costs. Similarly, genotypes with lower fruit density (higher RFV/RFM ratios) require greater storage volume and longer drying times, potentially limiting processing throughput during peak harvest periods. Thus, fruit physical traits directly influence not only yield but also logistics, processing efficiency, and the economic sustainability of coffee production systems (Dalazen et al., 2024; Ngure and Watanabe, 2024).

The present study addresses this gap by evaluating fruit physical characteristics and yield-related parameters of 44 *Coffea canephora* genotypes cultivated in eastern Minas Gerais, including both widely cultivated materials and novel farmer-selected genotypes. Specifically, we quantified RFM/DBM and RFV/RFM ratios, grain-to-husk proportion, and average yield in volume and mass. By integrating agronomic evaluation with indicators of processing efficiency, this study provides data directly relevant to breeding programs, industrial sourcing strategies, and regional planning, supporting the development of more economically efficient coffee production systems in emerging growing regions.

## 2 Materials and methods

### 2.1 Experimental area and cultivation conditions

The experiment was conducted in a commercial coffee plantation located on private property in the municipality of Aimorés, eastern Minas Gerais, Brazil. The site is located at 19°34′52″ S latitude, and 41°22′59″ W longitude, at an altitude of approximately 270 m above sea level. The predominant soil class in the study area is a Dystrophic Red-Yellow Latosol, corresponding to the Oxisol in USDA Soil Taxonomy and the Ferralsol in the World Reference Base classification (Santos et al., 2025).

The regional climate is classified as tropical savanna (Aw) according to the Köppen system, characterized by hot, humid summers and dry winters (Alvares et al., 2013; Beck et al., 2023). The area receives an average annual rainfall of approximately 887 mm and has a mean annual air temperature of 24.5 °C. Average minimum temperatures range from 16 to 22 °C and average maximum temperatures from 30 to 34 °C (Instituto Nacional de Meteorologia, 2025). During the two harvest seasons evaluated (2022/2023 and 2023/2024), monthly precipitation was concentrated between October and March, with peak values around 190 mm in December and January and was virtually absent from June to August (< 15 mm per month). Mean monthly air temperatures ranged from approximately 22 °C in July to 27 °C in January, with maximum temperatures reaching 32–33 °C during summer and minimum temperatures dropping to approximately 15 °C in the dry winter months, characterizing a pronounced seasonal contrast that defines Aimorés as a climatic frontier for *C. canephora* cultivation (Instituto Nacional de Meteorologia, 2025).

### 2.2 Plant material

A total of 44 *Coffea canephora* genotypes were evaluated. All genotypes were clonally propagated by stem cuttings (vegetative propagation) and cultivated at a spacing of 3.2 m between rows and 0.8 m between plants, totaling 3, 906 plants per hectare, each trained with two orthotropic stems. The genotype set comprised three groups of materials (**Table 1**): two Embrapa cultivars (category a), 23 promising Conilon genotypes originating from traditional producing regions in Espírito Santo (category c), and 19 seed-derived selections from eastern Minas Gerais (category b). These LMG genotypes (category b) were initially identified as seed-derived selections in farmers’ fields and were subsequently clonally propagated for inclusion in the trial. All genotypes were established simultaneously and evaluated at the same plant age. At the time of the evaluations, plants were approximately four years old, having been established in early 2020 and assessed across the 2022/2023 and 2023/2024 harvest seasons.

**Table 1:** Identification, and classification of the 44 *Coffea canephora* genotypes evaluated under field conditions in Aimorés, Minas Gerais, Brazil.

Identification	Cultivar reference	Identification	Cultivar reference
BRS 125^a^	Embrapa	CH1^c^	Farmer selection from Espírito Santo
BRS 88^a^	Embrapa	Imbugudinho^c^	Monte Pascoal^d^
LMG1^b^	Selection in eastern Minas Gerais	AD1^c^	Plena^d^
LMG2^b^	Selection in eastern Minas Gerais	Graudão HP^c^	Salutar^d^
LMG3^b^	Selection in eastern Minas Gerais	Valcir P^c^	Farmer selection from Espírito Santo
LMG4^b^	Selection in eastern Minas Gerais	Beira Rio 8^c^	Tributum^d^
LMG5^b^	Selection in eastern Minas Gerais	AP^c^	Monte Pascoal^d^
LMG6^b^	Selection in eastern Minas Gerais	L80^c^	Plena^d^
LMG7^b^	Selection in eastern Minas Gerais	Bamburral^c^	Tributum^d^
LMG8^b^	Selection in eastern Minas Gerais	Pirata^c^	Tributum^d^
LMG9^b^	Selection in eastern Minas Gerais	Peneirão^c^	Monte Pascoal and Plena^d^
LMG10^b^	Selection in eastern Minas Gerais	Ouro Negro^c^	Farmer selection from Espírito Santo
LMG11^b^	Selection in eastern Minas Gerais	A1^c^	Andina, Tributum, and Plena^d^
LMG12^b^	Selection in eastern Minas Gerais	P2^c^	Monte Pascoal^d^
LMG13^b^	Selection in eastern Minas Gerais	P1^c^	Andina^d^
LMG14^b^	Selection in eastern Minas Gerais	LB1^c^	Monte Pascoal and Plena^d^
LMG15^b^	Selection in eastern Minas Gerais	Clementino^c^	Tributum^d^
LMG16^b^	Selection in eastern Minas Gerais	Verdim TA ^c^	Andina^d^
LMG17^b^	Selection in eastern Minas Gerais	K61 ^c^	Farmer selection from Espírito Santo
LMG18^b^	Selection in eastern Minas Gerais	Guarani^c^	Forte Guarani^d^
LMG19^b^	Selection in eastern Minas Gerais	MP3^c^	Farmer selection
Bicudo ^c^	Plena ^d^	JN^c^	Farmer selection

^a^ Embrapa cultivars.

^b^ Seed-derived selections from eastern Minas Gerais (LMG genotypes).

^c^ Promising Conilon genotypes originating from traditional producing regions in Espírito Santo.

^d^ Partelli et al. (2021).

Crop management followed technical recommendations for *Coffea canephora* and was applied uniformly across all treatments (Ferrão et al., 2007; Vieira et al., 2005). Pruning was performed three to four times per year to maintain canopy structure and productive balance. Weed control was achieved through two to three annual applications of a systemic herbicide based on glyphosate. Phytosanitary management included preventive applications of fungicide and insecticide containing flutriafol and thiamethoxam, applied twice per year according to guidelines. Soil acidity was corrected by liming to raise base saturation to 70%. Fertilization was planned for an expected yield of 100–120–60 kg bags ha^−1^ year^−1^, with an annual nutrient supply of approximately 450 kg of N, 70 kg of P_2_O_5_, and 400 kg of K_2_O, applied through 12 fertigation events complemented by two additional topdressings.

The experiment was conducted using a randomized block design with 44 treatments (genotypes) and three replications (blocks). Each plot consisted of five plants, with evaluations performed on three central plants to minimize border effects. Assessments were carried out over two consecutive harvest seasons.

Plots were arranged in continuous rows within the commercial plantation to preserve the structural integrity of a real production system, ensuring that evaluated conditions reflect actual agronomic practice. The entire perimeter of the experiment was surrounded by border rows, providing external isolation from surrounding areas. Open pollination was not controlled, as *Coffea canephora* is a predominantly allogamous species and cross-pollination occurs naturally in commercial plantations. The fruit tissues evaluated in this study, including the pericarp and husk, are of maternal origin and primarily reflect the maternal genotype. Although the endosperm has a paternal contribution, the traits assessed here, RFM/DBM, RFV/RFM, and grain and husk mass percentages, are predominantly determined by maternal tissue development and fruit morphology and are therefore not expected to be substantially influenced by the pollen donor under the open-pollination conditions of this trial.

### 2.3 Sampling and evaluated variables

Harvesting was carried out when 80% of the fruits in each plot had reached the cherry stage, according to the maturation cycle of each genotype. All measurements were conducted at the plot level. Immediately after harvest, the ripe fruit volume (RFV) was measured using a graduated container, while the ripe fruit mass (RFM) was determined with a digital scale with a capacity of 150 kg.

From each plot, a random subsample of 20 cherry-stage fruits was collected for detailed yield analysis. This sample size was adopted based on previous studies evaluating fruit physical traits in *Coffea canephora*, in which subsamples of 20 fruits per plot have been shown to provide reliable estimates of grain and husk proportions while remaining operationally feasible in large-scale genotype trials (Partelli et al., 2021; Dalazen et al., 2024). The fruits were weighed, labeled, and stored in aerated containers prior to drying. Samples were dried in a forced-air oven at 50 ± 5 °C until reaching 12% moisture content. They were then weighed on a precision analytical balance and manually hulled to separate husk and beans. After hulling, the dry bean mass (DBM) and husk mass were individually measured for yield determination.

Based on these measurements, two yield-related ratios were calculated: the ratio between ripe fruit mass and dry bean mass (RFM/DBM; kg kg^−1^), and the ratio between ripe fruit volume and ripe fruit mass (RFV/RFM; L kg^−1^). The RFM/DBM ratio indicates how many kilograms of ripe fruits are required to obtain 1 kg of processed dry beans, whereas the RFV/RFM ratio reflects how many liters of ripe fruits correspond to 1 kg of ripe fruit mass. Based on these ratios, yield was also expressed as the volume and mass of ripe fruits required to produce a standard 60 kg bag of processed beans and as the volume per metric ton of processed beans, calculated as: fruit volume per bag (L bag^−1^) = RFV/RFM × RFM/DBM × 60; fruit mass per bag (kg bag^−1^) = RFM/DBM × 60; and fruit volume per metric ton (L t^−1^) = RFV/RFM × RFM/DBM × 1000, where 60 and 1000 correspond to the standard bag mass and metric ton in kilograms, respectively.

### 2.4 Statistical analysis

Prior to analysis, data were tested for compliance with the assumptions of analysis of variance. Residual normality was tested using the Shapiro-Wilk test (α = 0.05), and homogeneity of variances among genotypes was assessed by Bartlett’s test (α = 0.05). Subsequently, individual analyses of variance were performed for each harvest, followed by a combined analysis across harvests.

In the combined analysis, genotypes were considered as random effects, whereas year and block were treated as fixed effects, as the objective was to compare genotype performance across the specific harvest seasons evaluated. The genotype × year interaction was treated as a random effect. The adopted model was.


$$Y_{ijk}=\mu +G_{i}+A_{j}+B_{k}+{\left(GA\right)}_{ij}+\epsilon_{ijk}$$

where $Y_{ijk}$ represents the observation of the i-th genotype in the j-th year and the k-th block; μ is the overall mean; $G_{i}$ corresponds to the effect of the i-th genotype (random); $A_{j}$ is the fixed effect of the j-th year; $B_{k}$ is the effect of the k-th block, blocks were considered independent across years, as the experiment was conducted in a permanent commercial plantation where the same physical blocks were maintained across both harvest seasons. Block effects were therefore fitted as crossed with years rather than nested within years; ${\left(GA\right)}_{ij}$ is the genotype by year interaction (random); and $\epsilon_{ijk}$ is the experimental error. The significance of genotype, year, and genotype by year interaction effects was tested using the F-test at the 5% probability level.

For the RFM/DBM and RFV/RFM ratios, genetic ( $CV_{g}$) and experimental ( $CV_{e}$) coefficients of variation were calculated, expressed as percentages, according to the following formulas:


$$CV_{g}=(\frac{\sqrt{\frac{\left(MS_{g}-MS_{e}\right)}{r}}}{\bar{Y}})\times 100$$

$$CV_{e}=(\frac{\sqrt{MS}}{\bar{Y}})\times 100$$

where $QM_{g}$ is the mean square of genotypes, $MS_{e}$​ is the mean square of error, Y is the overall mean of the evaluated trait, and r is the number of replications. The ratio between coefficients of variation ( $CV_{g}/CV_{e}$) was also calculated. Broad-sense heritability (H²) was estimated from variance components according to the combined model, as:


$$H^{2}=\frac{\sigma_{g}^{2}}{\sigma_{g}^{2}+\frac{\sigma_{ga}^{2}}{a}+\frac{\sigma_{e}^{2}}{ar}}$$

where σg² is the genetic variance, σga² is the genotype × year interaction variance, σe² is the residual variance, a is the number of years (2), and r is the number of replications per year (3). This formulation accounts for the contribution of the genotype × year interaction to the uncertainty in genotype mean estimation. In addition, the intraclass correlation coefficient ( r) was calculated as:


$$r=\frac{\sigma_{g}^{2}}{\sigma_{g}^{2}+\sigma_{ga}^{2}+\sigma_{r}^{2}}$$

where $\sigma_{g}^{2}$ is the genetic variance, $\sigma_{ga}^{2}$ is the variance of genotype by year interaction, and $\sigma_{r}^{2}$ is the residual variance. Genotype means were compared using the Scott-Knott clustering test at the 5% probability level. Mean comparisons were performed on the adjusted genotype means (least squares means) obtained from the combined analysis. Although genotypes were treated as random effects for variance component estimation and heritability calculation, adjusted means were used for genotype discrimination and ranking, following standard practice in multi-harvest cultivar trials (Resende, 2007).

Phenotypic dissimilarity among the 44 genotypes was estimated based on the RFM/DBM and RFV/RFM ratios. Prior to analysis, variables were standardized to zero mean and unit variance. A dissimilarity matrix was constructed using Euclidean distance, calculated for each pair of genotypes i and j as:


$$d_{ij}=\sqrt{\sum {\left(x_{ik}-x_{jk}\right)}^{2}}$$

where $x_{ik}$ and $x_{jk}$ correspond to the values of the k-th variable for genotypes iand j.

Hierarchical clustering was performed using the UPGMA method (Unweighted Pair Group Method with Arithmetic Mean), which iteratively combines groups with the smallest average distance between their elements, preserving the hierarchical structure of similarity. The optimal number of groups was defined according to Mojena’s criterion (Mojena, 1977), adopting k=1.25, which provides objectivity in determining the dendrogram cut-off point. The consistency and quality of clustering were evaluated using the cophenetic correlation coefficient (CCC), calculated as the correlation between the original dissimilarity matrix and the cophenetic matrix derived from the dendrogram. Values closer to 1 indicate greater fidelity in representing the original distances.

Phenotypic dissimilarity among the 44 genotypes was estimated based exclusively on the RFM/DBM and RFV/RFM ratios, which represent the two central conversion efficiency indicators evaluated in this study.

All statistical analyses were performed using the GENES software (Cruz, 2016), employed for estimation of genetic parameters, clustering, and mean comparisons. Graphical representations, including the dendrogram and complementary graphs, was carried out in the R environment (R Core Team, 2025), using the *ggplot2* package (Wickham, 2009).

## 3 Results

### 3.1 Genetic parameters and experimental precision

The combined analysis of variance revealed significant differences among genotypes for both the ripe fruit mass to dry bean mass ratio (RFM/DBM; F = 2.431; p=0.002) and the ripe fruit volume to ripe fruit mass ratio (RFV/RFM; F = 1.933; p=0.017), while year effects were non-significant for both traits (**Table 2**). The genotype × year interaction was also non-significant (p=0.061 and p=0.408, respectively), indicating consistent genotype performance across the two harvest seasons evaluated. Mean RFM/DBM was 4.43 kg kg^−1^ in year 1 and 3.91 kg kg^−1^ in year 2, while mean RFV/RFM was 1.60 and 1.57 L kg^−1^, respectively. Broad-sense heritability (H^2^) was moderate to high, reaching 58.86% for the RFM/DBM ratio and 48.27% for the RFV/RFM ratio. The intraclass correlation coefficient indicated that genetic effects accounted for 22.89% of the total variance for RFM/DBM and 13.81% for RFV/RFM (**Table 3**).

**Table 2:** Combined analysis of variance for the ripe fruit mass to dry bean mass ratio (RFM/DBM) and the ripe fruit volume to ripe fruit mass ratio (RFV/RFM) of 44 *Coffea canephora* genotypes evaluated across two harvest seasons in Aimorés, Minas Gerais, Brazil.

Source of variation	df	MS (RFM/DBM)	F	p	MS (RFV/RFM)	F	p
Genotype (G)	43	0.7883	2.431	0.002	0.0254	1.933	0.017
Year (A)	1	0.0226	0.070	ns	0.0032	0.244	ns
G × A	43	0.3243	1.419	0.061	0.0131	1.045	0.408
Residual	176	0.2286	—	—	0.0126	—	—

†F-value for Genotype was tested against the G × A mean square; F-values for Year and G × A were tested against the Residual mean square. ns: not significant (p > 0.05).

**Table 3:** Estimates of genetic variance. ( $\sigma_{g}^{2}$), genotype × year interaction variance ( $\sigma_{ga}^{2}$), residual variance ( $\sigma_{e}^{2}$), broad-sense heritability ( $H^{2}$), intraclass correlation coefficient ( r), genetic ( $CV_{g}$) and experimental ( $CV_{e}$) coefficients of variation, and the $CV_{g}/CV_{e}$ratio, obtained for the RFM/DBM and RFV/RFM ratios.

Parameter	RFM/DBM (kg kg^−1^)	RFV/RFM (L kg^−1^)
$\sigma_{g}^{2}$	0.07733	0.00204
$\sigma_{ga}^{2}$	0.0319	0.00019
$\sigma_{e}^{2}$	0.22861	0.01255
$H^{2}$(%)	58.86	48.27
Intraclass correlation ( r)	22.89	13.81
$CV_{g}$	6.75	2.86
$CV_{e}$	11.64	7.15
$CV_{g}/CV_{e}$ ratio	0.58	0.40
Mean	4.13	1.58

The experimental coefficient of variation (CVe) was 11.64% for the relationship between ripe fruit mass (RFM) and dry bean mass (DBM), and 7.15% for the relationship between ripe fruit volume (RFV) and ripe fruit mass (RFM). Genetic coefficients of variation (CVg) exhibited distinct magnitudes: 6.75% for RFM/DBM and 2.86% for RFV/RFM, resulting in CVg/CVe ratios of 0.58 and 0.40, respectively. Overall means across genotypes were 4.13 kg kg^−1^ for RFM/DBM and 1.58 L kg^−1^ for RFV/RFM (**Table 3**).

### 3.2 Genotype clustering based on conversion efficiency

**Figure 1:**
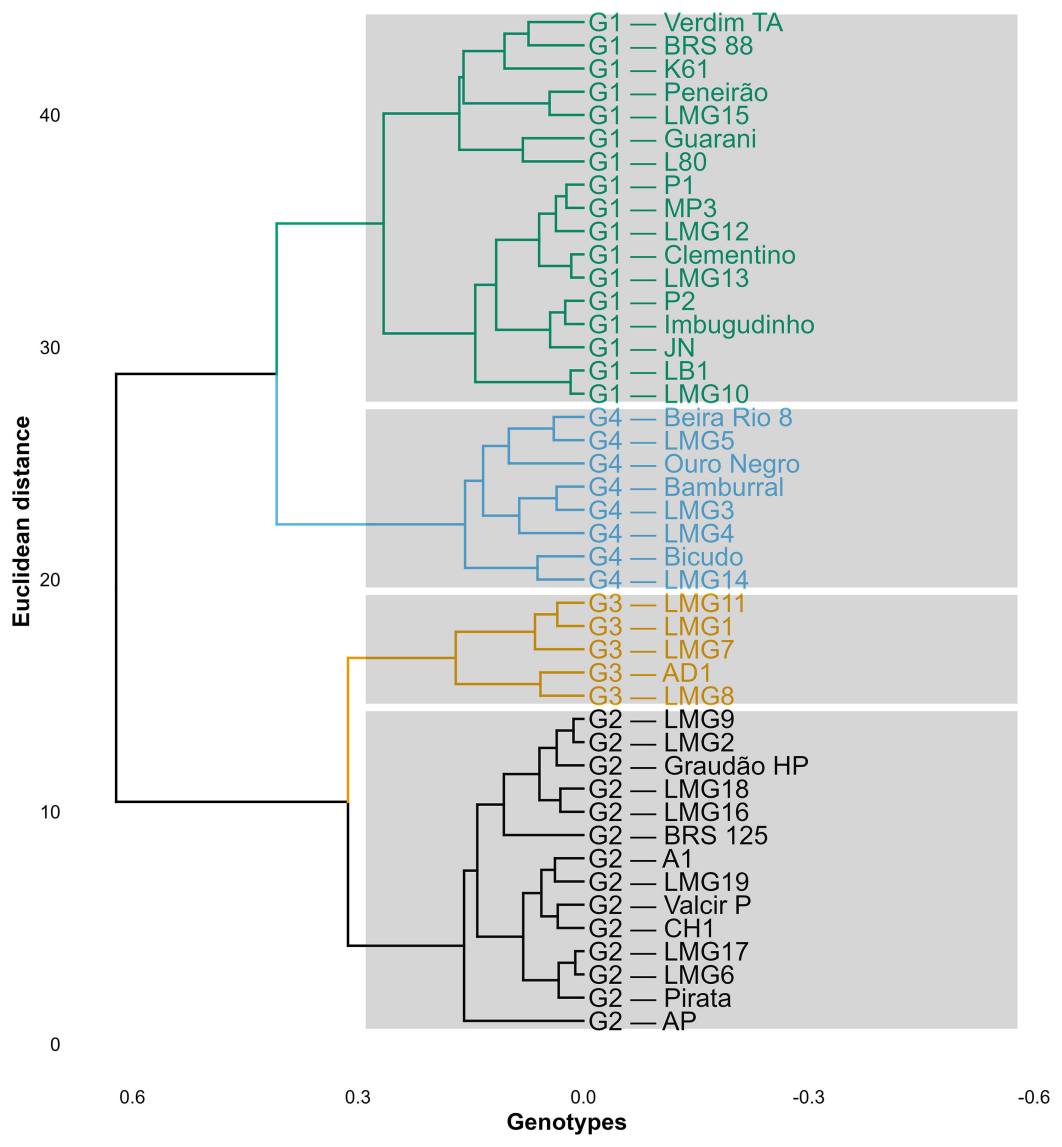
Dendrogram showing the hierarchical clustering of 44 *Coffea canephora* genotypes based on Euclidean distances calculated from the RFM/DBM and RFV/RFM ratios, using the UPGMA method. The four coloured groups (G1–G4) were defined by the cut-off point using Mojena’s criterion (k = 1.25). Genotypes are further identified by origin category, following the classification in **Table 1**: **(a)** Embrapa cultivars, **(b)** seed-derived selections from eastern Minas Gerais (LMG genotypes), **(c)** promising Conilon genotypes originating from traditional producing regions in Espírito Santo.

Hierarchical cluster analysis based on the RFM/DBM and RFV/RFM ratios identified four groups as the optimal partition, according to Mojena’s criteria. The cophenetic correlation coefficient was 0.73, a value that ensures good representativeness of the distance matrix by the dendrogram. The resulting dendrogram is presented in **Figure 1**.

Group 1 comprised established genotypes such as MP3, BRS 88, JN, Peneirão, Clementino, and Guarani, in addition to some experimental materials, characterized by higher RFM/DBM values, close to 4.3–4.4, and RFV/RFM values ranging from 1.53 to 1.69, indicating moderate fruit density and conversion efficiency. Group 2 included established genotypes such as BRS 125, Graudão HP, Pirata, and Valcir P, along with several LMG genotypes, showing lower RFM/DBM values (3.7–3.9) and RFV/RFM values around 1.56–1.66, reflecting comparatively higher efficiency in fruit-to-bean conversion.

Group 3 was predominantly composed of novel genotypes (LMG 1, LMG 7, LMG 8, LMG 11), in addition to AD1. This group presented the lowest RFM/DBM values among all clusters (3.48–3.66) and intermediate RFV/RFM values, indicating superior conversion efficiency with moderate fruit density. Group 4 comprised both novel genotypes from eastern Minas Gerais, LMG 3, LMG 4, LMG 5, and LMG 14, which belong to the group of seed derived selections (category b, **Table 1**), and previously studied materials from Espírito Santo, including Bicudo, Beira Rio 8, Bamburral, and Ouro Negro, classified as promising Conilon genotypes (category c, **Table 1**). This group was characterized by the highest RFM/DBM values (>4.6), indicating lower conversion efficiency, and RFV/RFM values ranging from 1.47 to 1.69, suggesting substantial variability in fruit density within the cluster.

### 3.3 Fruit-to-bean and fruit density ratios

**Figure 2:**
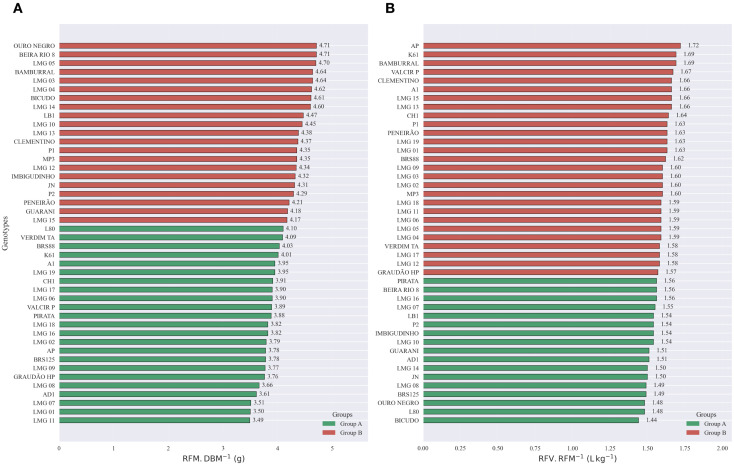
Mean values of the ripe fruit mass to dry bean mass ratio (RFM/DBM; kg kg^−1^) **(A)** and the ripe fruit volume to ripe fruit mass ratio (RFV/RFM; L kg^−1^) **(B)** for the 44 *Coffea canephora* genotypes. Bars represent the ordered genotype mean, and colors indicate the two groups formed by the Scott–Knott test at the 5% probability level (Group A and Group B). Numerical values correspond to the genotype means.

The RFM/DBM ratio displayed wide variation among genotypes, ranging from 3.49 kg kg^−1^ in LMG 11, a seed-derived selection from eastern Minas Gerais (category b, **Table 1**) to 4.71 kg kg^−1^ in Beira Rio 8 and Ouro Negro, both belonging to the group of promising Conilon materials from Espírito Santo (category c, **Table 1**). The Scott–Knott procedure separated the genotypes into two statistical groups. Beira Rio 8 (c), Ouro Negro (c), LMG 05 (b), and Bamburral (c) occupied the upper range of RFM/DBM, reflecting a greater mass of ripe fruits required to obtain 1 kg of processed dry beans. LMG 11 (b) and LMG 01 (b) formed the lower-ratio group, indicating more efficient conversion of fruit mass into dry beans (**Figure 2**).

The RFV/RFM ratio reinforced the structural contrasts observed among the genetic groups. Values extended from 1.44 L kg^−1^ in Bicudo, a representative of the Espírito Santo selections (category c, **Table 1**), to 1.72 L kg^−1^ in AP, also classified within this group (category c). Two clusters emerged from the statistical comparison. Bicudo (c), Ouro Negro (c), and L80 (c) exhibited lower RFV/RFM values, consistent with denser fruits requiring smaller volumes to reach 1 kg of ripe fruit mass. At the opposite end, AP (c), Bamburral (c), and K61 (c) showed higher ratios, reflecting less compact fruits and greater volumetric demand (**Figure 2**).

### 3.4 Grain and husk mass proportion

**Figure 3:**
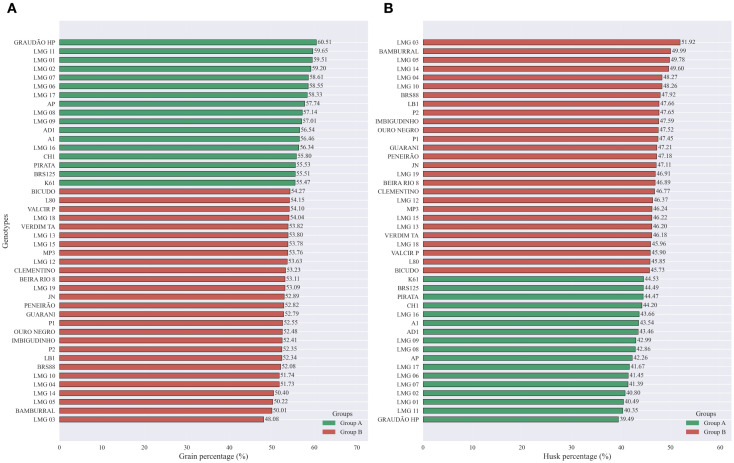
Grain **(A)** and husk **(B)** mass percentages of 44 *Coffea canephora* genotypes grouped by the Scott–Knott test at the 5% probability level. Bars represent genotype means adjusted to 12% moisture content. Identical color indicates that genotypes belong to the same statistical group, not differing significantly from one another.

*Coffea canephora* genotypes display broad variation in grain and husk allocation. Grain percentages extended from 48.08 percent in LMG 03, a seed-derived selection from eastern Minas Gerais (category b, **Table 1**) to 60.51 percent in Graudão HP, a genotype originating from Espírito Santo (category c, **Table 1**). The Scott–Knott test separated the materials into two sets. The upper group included Graudão HP (c), LMG 11 (b), LMG 01 (b), and LMG 02 (b), all exhibiting grain proportions equal to or above 55.47 percent. The second group comprised genotypes with lower grain percentages and, consequently, higher husk proportions, such as LMG 03 (b), Bamburral (c), and LMG 05 (b). The contrast between these sets’ underscores consistent differences in the distribution of fruit biomass among the evaluated materials (**Figure 3**).

The pattern for husk percentage mirrored this structure. Values ranged from 39.49 percent in Graudão HP (c) to 51.92 percent in LMG 03 (b). Genotypes in the high-grain group exhibited lower husk proportions, reflecting more efficient fruit utilization, whereas those in the low-grain group showed higher husk percentages, indicative of reduced yield in processed beans. The observed dispersion confirms the presence of phenotypic variability in grain and husk allocation and supports the relevance of these traits as selection criteria in breeding programs (**Figure 3**).

### 3.5 Yield expressed as fruit volume and mass

**Figure 4:**
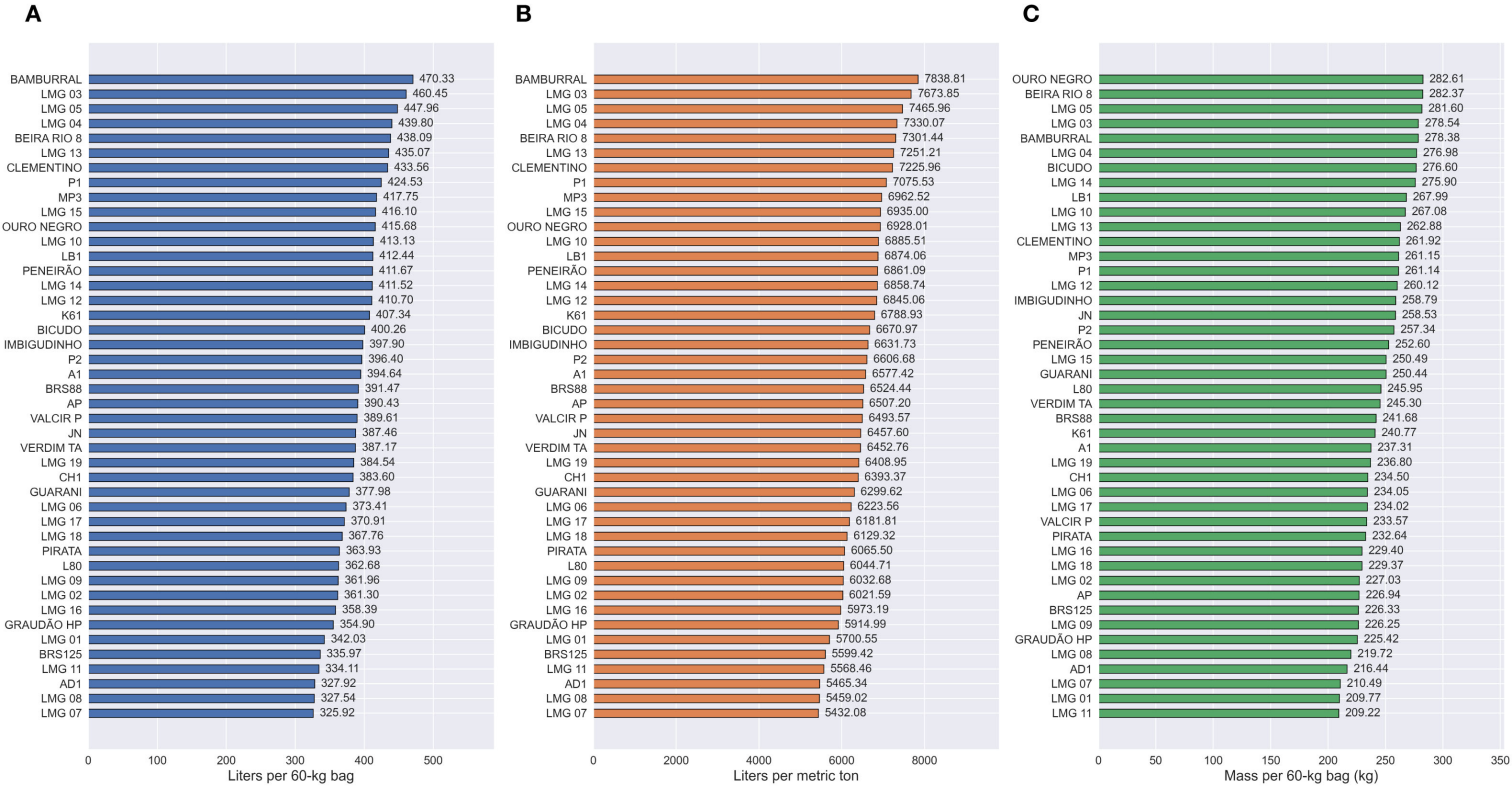
Mean yield of 44 *Coffea canephora* genotypes evaluated in Aimorés, Minas Gerais, considering: **(a)** mean volume of ripe fruits (L) required to obtain a 60-kg bag of processed beans; **(b)** mean volume of ripe fruits (L) per metric ton of processed beans; and **(c)** mean mass of ripe fruits (kg) per 60-kg bag of processed beans. Bars represent means adjusted to 12% moisture content.

Substantial variation emerged among the 44 *Coffea canephora* genotypes in yield expressed as fruit volume and mass. The volume of ripe coffee required to obtain a 60 kg bag of processed beans ranged from 325.92 L in LMG 07, a seed-derived selection from eastern Minas Gerais (category b, **Table 1**) to 470.33 L in Bamburral, a genotype originating from Espírito Santo (category c, **Table 1**), an amplitude of 44.31 percent. When expressed as fruit mass, values extended from 209.22 kg in LMG 11 (b) to 282.61 kg in Ouro Negro (c), corresponding to a 35.08 percent variation among genotypes (**Figure 4**).

The overall mean across genotypes was 391.28 L per 60 kg bag, 6, 037.51 L per metric ton of processed beans, and 247.19 kg of ripe coffee per 60 kg bag. Several materials required less than 350 L of ripe fruits to produce a processed bag, including Graudão HP, a promising genotype from Espírito Santo (category c, **Table 1**), and the LMG selections LMG 07 (b), LMG 08 (b), and LMG 11 (b). At the opposite end, Bamburral (c), LMG 03 (b), and LMG 05 (b) exceeded 440 L, revealing marked contrasts in fruit-to-bean conversion efficiency among the evaluated materials (**Figure 4**).

## 4 Discussion

Overall, the results indicate that several novel genotypes exhibited performance comparable to widely cultivated commercial materials for fruit-to-bean conversion efficiency. However, these findings are based on a two-year evaluation in a single location, and additional multi-environment trials are needed to confirm stability across diverse conditions. This finding indicates that regional germplasm from eastern Minas Gerais harbors exploitable variability relevant for breeding programs and commercial production systems. The consistency of genetic parameters across consecutive harvests further suggests that the observed differences are stable and potentially heritable, reflecting stable genotypic contrasts.

The evaluation of 44 *Coffea canephora* genotypes revealed significant genetic variability for RFM/DBM and RFV/RFM ratios, grain and husk mass percentages, and yield in volume and mass. This variability represents a fundamental prerequisite for the success of breeding programs, as it expands the range of selectable phenotypes and supports targeted recombination strategies aimed at improving processing efficiency and resource use (Silva et al., 2025; Sousa et al., 2025).

Although narrow−sense heritability usually guides selection decisions, broad−sense estimates still offer value in perennial crops during early screening (Resende and Alves, 2020). They incorporate non−additive components, which limit their predictive power for genetic gain, but they do capture the extent to which phenotypic variation is genetically structured. Under this lens, the heritability values observed here suggest that fruit−to−bean conversion efficiency has enough genetic determination to justify further selection efforts. In this context, the observed heritability values indicate that a meaningful proportion of the phenotypic variation in fruit-to-bean conversion efficiency is genetically determined and therefore exploitable in selection programs.

Intraclass correlation coefficients were moderate, reflecting the combined influence of environmental variability and genotype × year interaction, which is expected in multi-year field experiments. Nevertheless, the coexistence of moderate heritability and significant phenotypic variation provides a consistent basis for discriminating among genotypes (Resende, 2007). Similar patterns have been reported in other *C. canephora* populations evaluated across contrasting environments, reinforcing the robustness of these traits as selection targets (Alkimim et al., 2021).

Experimental coefficients of variation were low (11.64% for RFM/DBM and 7.15% for RFV/RFM), indicating good experimental precision (Cargnelutti Filho and Storck, 2007; Pimentel-Gomes, 2008). Genetic coefficients of variation were reduced (6.75% and 2.86%), resulting in CV_g/CV_eratios of 0.40 and 0.58. Since ratios below 1.0 indicate that environmental variance exceeds genetic variance (Faleiro et al., 2002), these results reflect the experimental precision of the trial and the magnitude of environmental effects relative to genetic differentiation under the specific conditions evaluated. Despite the modest CVg/CVe ratios, significant genetic variation was detected (p < 0.05), and the phenotypic ranges observed confirm the presence of exploitable genetic differences among genotypes. Even so, the phenotypic ranges observed confirm the presence of measurable differences among genotypes. Given the high agronomic and industrial relevance of fruit-to-bean conversion traits, even modest genetic gains may translate into cumulative improvements in processing efficiency over successive selection cycles. Moreover, genetic diversity in these traits may also be associated with physiological and nutritional efficiency, which are critical for genotype adaptation in emerging production environments (da Silva et al., 2025).

These results align with global trends observed in emerging cultivation regions for *C. canephora*, such as parts of East Africa and Southeast Asia, where the species’ genetic plasticity has enabled the expansion of productive areas under increasingly variable climatic conditions (Ngure and Watanabe, 2024). Thus, the identification of adaptable genotypes in transitional environments reinforces Brazil’s strategic role in the conservation and global improvement of this crop.

The hierarchical clustering analysis revealed four distinct groups, highlighting structured variability among genotypes. Group 1 was characterized by higher RFM/DBM values, indicating lower conversion efficiency, which is consistent with previous reports for widely cultivated materials (Ferrão et al., 2024). In contrast, other clusters combined lower RFM/DBM values with moderate RFV/RFM values, reflecting improved efficiency in converting fruit biomass into processed beans. Group 2 comprised genotypes already studied that consistently show higher productive efficiency, along with several LMG genotypes that aligned with this profile. The presence of both established and novel genotypes within the same efficiency-oriented clusters suggests that part of the regional germplasm shares desirable processing traits already observed in consolidated materials (Partelli et al., 2021).

Group 3, dominated by novel genotypes exhibiting the lowest RFM/DBM values, suggests the presence of genetic variability that warrants further evaluation in breeding programs. However, the agronomic performance and industrial viability of these materials require validation under diverse environmental conditions and commercial-scale processing systems before definitive recommendations can be made. Conversely, the clustering of some novel materials with less efficient genotypes in Group 4 highlights the heterogeneity of farmer-selected germplasm and underscores the importance of systematic evaluation prior to recommendation (Ngure and Watanabe, 2024).

Variation in RFM/DBM and RFV/RFM ratios futher confirmed that fruit-to-bean conversion efficiency and fruit density are complementary traits capable of discriminating genotypes. For the RFM/DBM ratio, lower values, such as those observed in LMG 11 and LMG 01, indicate greater efficiency in converting ripe fruits into processed beans, a desirable trait in breeding programs as it reflects higher yield. Conversely, genotypes such as Beira Rio 8, Ouro Negro, LMG 05, and Bamburral exhibited higher values, implying that a larger quantity of ripe fruits is required to obtain 1 kg of processed coffee. The joint consideration of these traits therefore provides a more comprehensive assessment of processing efficiency than either indicator alone.

The RFV/RFM ratio also revealed two distinct clusters. Genotypes such as Bicudo, Ouro Negro, and L80 displayed lower values, associated with denser fruits, a condition that reflects a higher proportion of beans relative to husk and therefore greater fruit utilization. In contrast, materials such as AP, Bamburral, and K61 exhibited higher values, suggesting less compact fruits, which may require greater harvest and transport volume for the same amount of processed beans, in addition to highlighting physiological differences among genotypes.

The separation of genotypes into statistically distinct groups for both ratios reinforces the existence of exploitable variability and supports selection strategies that integrate biomass conversion and fruit physical characteristics. Such an approach is particularly relevant in emerging production areas, where processing infrastructure and logistics may amplify the economic impact of differences in fruit volume and mass requirements.

The wide variation observed in grain (48.08% to 60.51%) and husk mass (39.49% to 51.92%) further support the presence of genetic differences in fruit biomass partitioning. Genotypes with higher grain proportions and lower husk percentages exhibited more efficient utilization of fruit biomass, and included Graudão HP and LMG 11, belonging to group “A, “. In contrast, genotypes such as LMG 03 and Bamburral, in group “B, “ showed higher husk proportions implying reduced efficiency (Bezerra et al., 2023). These differences have direct implications for processing yield, as genotypes with lower grain percentages may require 20–30% more fruit volume and mass to produce a processed bag, reinforcing their reduced productive efficiency (Rocha et al., 2021).

The consistency between grain percentage, RFM/DBM, and RFV/RFM results reinforces the internal coherence of the evaluated indicators. Genotypes combining higher grain proportions with lower RFM/DBM and RFV/RFM values consistently required less fruit volume and mass to produce processed beans, highlighting the relevance of integrating these traits in selection schemes.

Yield expressed as fruit volume and mass revealed substantial contrasts among *Coffea canephora* genotypes, reflecting substantial differences in the efficiency of converting ripe fruits into processed coffee. While genotypes such as LMG 07, LMG 08, and LMG 11 required less than 350 L of fruits to obtain a 60 kg bag, others such as Bamburral, LMG 03, and LMG 05 exceeded 440 L, reflecting lower yield. This variation, which reached 44.31% in volume and 35.08% in mass, is directly related to biomass accumulation in different fruit components, particularly the proportion of beans and husk. In practical terms, genotypes with lower grain percentages may require 20–30% more fruit biomass to produce a processed bag, reducing efficiency. Moreover, they may demand greater nutrient input to produce one metric ton of processed coffee. Differences in fruit volume and mass requirements are particularly relevant at industrial scale, where drying capacity, storage volume, and processing throughput often represent critical bottlenecks. Genotypes requiring lower fruit inputs per unit of processed coffee may therefore contribute to improved efficiency and reduced operational constraints in centralized processing facilities.

The mean values observed in Aimorés were consistent with those reported for traditional producing regions in Brazil such as Espírito Santo (Dalazen et al., 2024; Partelli et al., 2021) and Rondônia (Schmidt et al., 2023), confirming the consistency of these parameters as yield indicators. This performance range is also comparable to that observed in African clones of *C. canephora*, where differences in vigor and productivity also reflect high genetic variability (Akpertey et al., 2022). Nevertheless, morphological traits such as fruit size, shape, and the occurrence of peaberries likely contributed to the observed variability, emphasizing that yield is the outcome of complex interactions between genotype and cultivation conditions.

The results presented here should be interpreted within the context of their experimental limitations. The evaluation was conducted in a single location over two consecutive harvest seasons, a design that reveals genotype differentiation but does not capture genotype × environment interactions across broader climatic and edaphic gradients. Commercial scale validation is also necessary to assess processing performance under industrial conditions, since fruit handling, drying procedures, and storage duration may influence the realized conversion efficiency. Additionally, the relationship between fruit to bean conversion traits and other agronomically relevant characteristics, such as total yield, vegetative vigor, disease resistance, and long-term productivity, requires integrated evaluation to support breeding decisions.

Despite these limitations, the results provide a foundation for further research and contribute to the characterization of regional germplasm in an emerging coffee production frontier. Future work should prioritize validation trials across contrasting environments, integration with molecular breeding tools, and assessment of processing performance under commercial conditions. Such efforts will be critical to consolidate these findings and support the deployment of improved genetic materials in climate resilient production systems.

## 5 Conclusions

This study detected significant genetic variability among 44 *Coffea canephora* genotypes cultivated in Aimorés, Minas Gerais, Brazil, evaluated across two consecutive harvest seasons. Differences were observed in fruit-to-bean conversion efficiency, fruit density, grain and husk percentages, and yield expressed in volume and mass. However, these findings are based on a single-location trial and require validation across diverse environments and production systems before broader recommendations can be made.

Several materials showed favorable performance across the evaluated traits. Among the seed-derived selections from eastern Minas Gerais (category b), LMG 01, LMG 07, LMG 08, and LMG 11 exhibited the most favorable conversion efficiency, combining lower RFM/DBM ratios, higher grain mass percentages, and reduced fruit volume requirements per processed bag. Among the established genotypes from Espírito Santo (category c), Graudão HP stood out for its high grain proportion (60.51%) and low fruit volume requirement per bag. These materials represent candidates for further evaluation in breeding programs and multi-environment trials aimed at developing commercial recommendations.

The results help clarify the genetic landscape of this emerging coffee−growing region and offer useful baseline parameters for comparison. Although the single−location and short−term design limit the strength of agronomic inferences, the patterns observed point to materials with potential relevance for efficiency−oriented production systems. Broader validation across environments, harvest cycles, and processing conditions, together with integration of key agronomic traits, will be necessary to translate these findings into practical breeding and deployment decisions.

## Data Availability

The raw data supporting the conclusions of this article will be made available by the authors, without undue reservation.
